# Source of Funding and Results of Studies of Health Effects of Mobile Phone Use: Systematic Review of Experimental Studies

**DOI:** 10.1289/ehp.9149

**Published:** 2006-09-15

**Authors:** Anke Huss, Matthias Egger, Kerstin Hug, Karin Huwiler-Müntener, Martin Röösli

**Affiliations:** 1 Department of Social and Preventive Medicine, University of Berne, Berne, Switzerland; 2 Department of Social Medicine, University of Bristol, United Kingdom; 3 Institute of Social and Preventive Medicine, University of Basle, Basle, Switzerland

**Keywords:** electromagnetic fields, financial conflicts of interest, human laboratory studies, mobile phones

## Abstract

**Objectives:**

There is concern regarding the possible health effects of cellular telephone use. We examined whether the source of funding of studies of the effects of low-level radiofrequency radiation is associated with the results of studies. We conducted a systematic review of studies of controlled exposure to radiofrequency radiation with health-related outcomes (electroencephalogram, cognitive or cardiovascular function, hormone levels, symptoms, and subjective well-being).

**Data sources:**

We searched EMBASE, Medline, and a specialist database in February 2005 and scrutinized reference lists from relevant publications.

**Data extraction:**

Data on the source of funding, study design, methodologic quality, and other study characteristics were extracted. The primary outcome was the reporting of at least one statistically significant association between the exposure and a health-related outcome. Data were analyzed using logistic regression models.

**Data synthesis:**

Of 59 studies, 12 (20%) were funded exclusively by the telecommunications industry, 11 (19%) were funded by public agencies or charities, 14 (24%) had mixed funding (including industry), and in 22 (37%) the source of funding was not reported. Studies funded exclusively by industry reported the largest number of outcomes, but were least likely to report a statistically significant result: The odds ratio was 0.11 (95% confidence interval, 0.02–0.78), compared with studies funded by public agencies or charities. This finding was not materially altered in analyses adjusted for the number of outcomes reported, study quality, and other factors.

**Conclusion:**

Conclusions: The interpretation of results from studies of health effects of radiofrequency radiation should take sponsorship into account.

The use of mobile telephones has increased rapidly in recent years. The emission of low-level radiofrequency electromagnetic fields leading to the absorption of radiation by the brain in users of handheld mobile phones has raised concerns regarding potential effects on health (Rothman 2000). However, the studies examining this issue have produced conflicting results, and there is ongoing debate on this issue (Ahlbom et al. 2004; Feychting et al. 2005). Many of the relevant studies have been funded by the telecommunications industry, and thus may have resulted in conflicts of interest (Thompson 1993). Recent systematic reviews of the influence of financial interests in medical research concluded that there is a strong association between industry sponsorship and pro-industry conclusions (Bekelman et al. 2003; Yaphe et al. 2001). This association has not been examined in the context of the studies of potential adverse effects of mobile phone use. We performed a systematic review and analysis of the literature to examine whether industry involvement is associated with the results and methodologic quality of studies.

## Methods

We searched EMBASE (http://www.embase.com) and Medline (http://www.ncbi.nlm.nih.gov/entrez/query.fcgi?DB=pubmed) in February 2005. Key and free text words included “cell(ular),” “mobile,” “(tele)phone(s)” in connection with “attention,” “auditory,” “bioelectric,” “brain physiology,” “cardiovascular,” “cerebral,” “circulatory,” “cognitive,” “EEG,” “health complaint(s),” “hearing,” “heart rate,” “hormone(s),” “learning,” “melatonin,” “memory,” “neural,” “neurological,” “nervous system,” “reaction,” “visual,” “symptom(s),” or “well-being.” The search was complemented with references from a specialist database (ELMAR 2005) and by scrutinizing reference lists from the relevant publications. Articles published in English, German, or French were considered.

We included original articles that reported studies of the effect of controlled exposure with radiofrequency radiation on health-related outcomes [“human laboratory studies” in World Health Organization (WHO) terminology (Repacholi 1998)]. Health-related outcomes included electroencephalogram (EEG) recordings, assessments of cognitive or cardiovascular function, hormone levels, and subjective well-being and symptoms. We excluded studies of the risk of using mobile phones when driving a motor vehicle or operating machinery as well as studies on electromagnetic field (EMF) incompatibilities (e.g., pacemakers or hearing aids). Three of us (A.H., K.H., M.R.) independently extracted data on the source of funding (industry, public or charity, mixed, not reported) and potential confounding factors, including study design (crossover, parallel, other), exposure (frequency band, duration, field intensity, and location of antenna), and methodologic and reporting quality. Four dimensions of quality were assessed (Jüni et al. 2001; Repacholi 1998): *a*) randomized, concealed allocation of study participants in parallel or crossover trials; *b*) blinding of participants and investigators to allocation group; *c*) reporting of the specific absorption rate (SAR; watts per kilogram tissue) from direct measurement using a phantom head or three-dimensional dosimetric calculations (“appropriate exposure setting”); *d*) appropriate statistical analysis. For each item, studies were classified as adequate or inadequate/unclear.

The primary outcome was the reporting of at least one statistically significant (*p* < 0.05) association between radiofrequency exposure and a health-related outcome. The message in the title was also assessed. We distinguished among neutral titles [e.g., “Human brain activity during exposure to radiofrequency fields emitted by cellular phones” (Hietanen et al. 2000)], titles indicating an effect of radiation [e.g., “Exposure to pulsed high-frequency electromagnetic field during waking affects human sleep EEG” (Huber et al. 2000)], and titles stating that no effect was shown [e.g., “No effect on cognitive function from daily mobile phone use” (Besset et al. 2005)]. Finally, authors’ declaration of conflicts of interest (present, absent) and affiliations (industry, other) were recorded. Differences in data extracted by A.H., K.H., and M.R. were resolved in the group, with the senior epidemiologist (M.R.) acting as the arbiter. In addition, two of us (K.H.M., M.E.), who were kept blind to funding source, authors, and institutions, repeated extraction of data from abstracts and assessments of titles. Differences in data extracted by K.H.M. and M.E. were resolved with the senior epidemiologist (M.E.) acting as the arbiter. Based on the abstracts, we assessed whether authors interpreted their study results as showing an effect of low-level radiofrequency radiation, as showing no effect, or as indicating an unclear finding.

We used logistic regression models to assess whether the source of funding was associated with the reporting of at least one significant effect in the article (including the abstract). We examined the influence of potential confounders, such as the total number of outcomes that were reported in the article, the type of study (crossover, parallel, other), the four dimensions of study quality (adequate or not adequate/unclear), exposure conditions (position of the antenna next to the ear compared with other locations; use of the 900-MHz band compared with other bands; duration of exposure in minutes), as well as the type of outcome (e.g., cognitive function tests: yes vs. no). Variables were entered one at a time and, given the limited number of studies, models were adjusted for one variable only. Results are reported as odds ratios (ORs) with 95% confidence intervals (CIs). All analyses were carried out in Stata (version 8.2; StataCorp., College Station, TX, USA).

## Results

**Figure 1:**
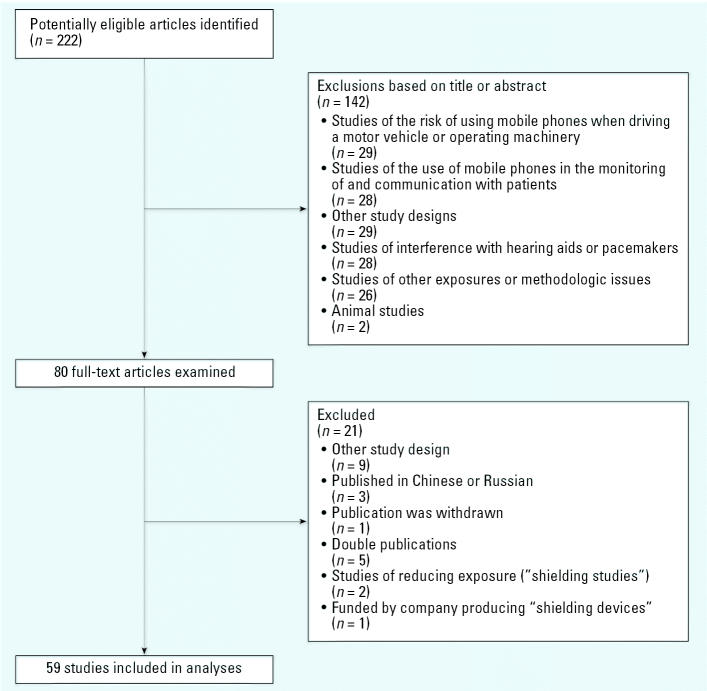
Identification of eligible studies.

We identified 222 potentially relevant publications and excluded 163 studies that did not meet inclusion criteria (Figure 1). We excluded one study that had been funded by a company producing “shielding” devices that reduce EMF exposure (Croft et al. 2002). A total of 59 studies were included: 12 (20%) were exclusively funded by the telecommunications industry, 11 (19%) were funded by public agencies or charities, 14 (24%) had mixed funding (including industry and industry-independent sources), and in 22 (37%) studies the source of funding was not reported. None of 31 journals published a statement on possible conflicts of interest of the 287 authors listed in the bylines. Five (8%) studies had authors with industry affiliation. All studies except two (3%) were published in journals that use peer review, and one was published in a journal supplement. The bibliographic references are given in the Supplemental Material (http://www.ehponline.org/members/2006/9149/supplemental.pdf).

Blinded and open extraction of data yielded identical results with respect to the reporting of statistically significant effects in the abstract and the message of the title. Study characteristics are shown in Table 1. All studies were published during 1995–2005, with the number of publications increasing from one to two publications per year to 11 publications in 2004. Median year of publication was 1998 for industry-funded studies, 2002 for public or charity funding and studies with mixed funding sources, and 2003 for studies that did not report their funding source. The median size of all the studies was small (20 study participants); most studies (*n* = 32, 54%) were of a crossover design and mimicked the exposure situation during a phone call, using the 900-MHz band with the antenna located close to the ear. Exposure duration ranged from 3 to 480 min, with a median of 33 minutes. Thirty-three (59%) studies measured outcomes during exposure, 14 (24%) postexposure, and 12 (20%) at both times. Thirty-nine (66%) studies prevented selection bias with adequate randomization; 15 (25%) blinded both participants and assessors; in 18 (31%) the field intensity had been assessed appropriately, with SAR values ranging from 0.03 to 2 W/kg tissue. Finally, in 14 (24%) studies we considered the statistical analysis to be adequate. Study quality varied by source of funding: Studies with mixed funding (including public agencies or charities and industry) had the highest quality, whereas studies with no reported source of funding did worst (Table 1).

Forty (68%) studies reported one or more statistically significant results (*p* < 0.05) indicating an effect of the exposure (Table 2). Studies funded exclusively by industry reported on the largest number of outcomes but were less likely to report statistically significant results: The OR for reporting at least one such result was 0.11 (95% CI, 0.02–0.78), compared with studies funded by public agencies or charities (Table 3). This finding was not materially altered in analyses adjusted for the number of outcomes reported, study design and quality, exposure characteristics, or outcomes [Table 3; see Supplemental Material, Table 1 (http://www.ehponline.org/members/2006/9149/supplemental.pdf)]. Similar results were obtained when restricting analyses to results reported in abstracts (OR = 0.29; 95% CI, 0.05–1.59) or on the conclusions in the abstract (OR = 0.10, 95% CI, 0.009–1.10). Thirty-seven (63%) studies had a neutral title, 11 (19%) a title reporting an effect, and 11 (19%) a title reporting no effect (Table 2).

## Discussion

We examined the methodologic quality and results of experimental studies investigating the effects of the type of radiofrequency radiation emitted by handheld cellular telephones. We hypothesized that studies would be less likely to show an effect of the exposure if funded by the telecommunications industry, which has a vested interest in portraying the use of mobile phones as safe. We found that the studies funded exclusively by industry were indeed substantially less likely to report statistically significant effects on a range of end points that may be relevant to health.

Our findings add to the existing evidence that single-source sponsorship is associated with outcomes that favor the sponsors’ products (Bekelman et al. 2003; Davidson 1986; Lexchin et al. 2003; Stelfox et al. 1998). Most previous studies of this issue were based on studies of the efficacy and cost-effectiveness of drug treatments. A recent systematic review and meta-analysis showed that studies sponsored by the pharmaceutical industry were approximately four times more likely to have outcomes favoring the sponsor’s drug than studies with other sources of funding (Lexchin et al. 2003). The influence of the tobacco industry on the research it funded has also been investigated (Barnes and Bero 1996, 1998; Bero 2005). To our knowledge, this is the first study to examine this issue in the context of exposure to radiofrequency electromagnetic fields.

Our study has several limitations. We restricted our analysis to human laboratory studies. This resulted in a more homogenous set of studies, but may have reduced the statistical power to demonstrate or exclude smaller associations. The WHO has identified the need for further studies of this type to clarify the effects of radiofrequency exposure on neuroendocrine, neurologic, and immune systems (Foster and Repacholi 2004). We considered including epidemiologic studies but found that practically all of them were publicly funded. The study’s primary outcome—the reporting of statistically significant associations—is a crude measure that ignores the size of reported effects. However, we found the same trends when assessing the authors’ conclusions in the abstracts.

Although we have shown an association between sponsorship and results, it remains unclear which type of funding leads to the most accurate estimates of the effects of radiofrequency radiation. For example, if researchers with an environmentalist agenda are more likely to be funded by public agencies or charities, then their bias may result in an overestimation of effects. Interestingly, studies with mixed funding were of the highest quality. The National Radiological Protection Board (NRPB 2004) reviewed studies of health effects from radiofrequency (RF) fields and concluded that “scientific evidence regarding effects of RF field exposure from mobile phones on human brain activity and cognitive function … has included results both supporting and against the hypothesis of an effect.” We found that the source of funding explains some of the heterogeneity in the results from different studies. The association was robust and little affected by potential confounding factors such as sample size, study design, or quality.

Possible explanations for the association between source of funding and results have been discussed in the context of clinical research sponsored by the pharmaceutical industry (Baker et al. 2003; Bekelman et al. 2003; Lexchin et al. 2003). The association could reflect the selective publication of studies that produced results that fitted the sponsor’s agenda. Sponsors might influence the design of the study, the nature of the exposure, and the type of outcomes assessed. In multivariate logistic regression analysis, the only factor that strongly predicted the reporting of statistically significant effects was whether or not the study was funded exclusively by industry. We stress that our ability to control for potential confounding factors may have been hampered by the incomplete reporting of relevant study characteristics.

Medical and science journals are implementing policies that require authors to disclose their financial and other conflicts of interest. None of the articles examined here included such a statement, in line with a survey of science and medical journals that showed that adopting such policies does not generally lead to the publication of disclosure statements (Krimsky and Rothenberg 2001). A review of 2005 instructions to authors showed that 15 (48%) of the 31 journals included in our study had conflict of interest policies. Our results support the notion that disclosure statements should be published, including statements indicating the absence of conflicts of interest. The role of the funding source in the design, conduct, analysis, and reporting of the study should also be addressed.

There is widespread concern regarding the possible health effects associated with the use of cellular phones, mobile telephone base stations, or broadcasting transmitters. Most (68%) of the studies assessed here reported biologic effects. At present it is unclear whether these biologic effects translate into relevant health hazards. Reports from national and international bodies have recently concluded that further research efforts are needed, and dedicated research programs have been set up in the United States, Germany, Denmark, Hungary, Switzerland, and Japan. Our study indicates that the interpretation of the results from existing and future studies of the health effects of radiofrequency radiation should take sponsorship into account.

## Figures and Tables

**Table 1:** Characteristics of 59 experimental studies of the effects of exposure to low-level radiofrequency electromagnetic fields.

	Source of funding			
Study characteristic	Industry (*n* = 12)	Public or charity (*n* = 11)	Mixed (*n* = 14)	Not reported (*n* = 22)
Study design [no. (%)]				
Crossover trial	10 (83.3)	7 (63.6)	12 (85.7)	11 (50)
Parallel group trial	0 (0)	2 (18.2)	1 (7.1)	2 (9.1)
Other, unclear	2 (16.7)	2 (18.2)	1 (7.1)	9 (40.9)
Exposure [no. (%)]				
Location of antenna				
Next to ear	4 (33.3)	8 (72.7)	11 (78.6)	14 (63.6)
Other/unclear	8 (66.7)	3 (27.3)	3 (21.4)	8 (36.4)
Frequency band^a^				
900 MHz	11 (91.7)	8 (72.7)	13 (92.9)	14 (63.6)
Other frequencies	2 (16.7)	7 (63.6)	0 (0)	5 (22.7)
Unclear	0 (0)	0 (0)	1 (7.1)	5 (22.7)
Median duration of exposure (range)	180 (3–480)	20 (5–35)	45 (30–240)	30 (4–480)
Outcomes assessed [no. (%)]^a^				
Electroencephalogram	7 (58.3)	5 (45.5)	8 (57.1)	12 (54.5)
Cognitive function tests	0 (0)	3 (27.3)	8 (57.1)	8 (36.4)
Hormone levels	5 (41.7)	0 (0)	0 (0)	2 (9.1)
Cardiovascular function	2 (16.7)	1 (9.1)	0 (0)	2 (9.1)
Well-being or symptoms	1 (8.3)	1 (9.1)	1 (7.1)	0 (0)
Other	4 (33.3)	3 (27.3)	1 (7.1)	3 (13.6)
Study quality [no. (%)]^a^				
Randomization adequate	10 (83.3)	7 (63.6)	13 (92.9)	9 (40.9)
Participants and assessors blinded	1 (8.3)	3 (27.3)	8 (57.1)	3 (13.6)
SAR determined	4 (33.3)	4 (36.4)	8 (57.1)	2 (9.1)
Statistical analysis adequate	3 (25)	3 (27.3)	7 (50)	1 (4.5)
Median study size (range)	21 (8–38)	24 (13–100)	20 (13–96)	20 (8–78)

Percentages are column percentages.

a The same study could be listed in more than one category.

**Table 2:** Results from assessments of article text, abstract, and title of 59 experimental studies of the effects of exposure to low-level radiofrequency electromagnetic fields.

	Source of funding			
	Industry (*n* = 12)	Public or charity (*n* = 11)	Mixed (*n* = 14)	Not reported (*n* = 22)
Article text				
No. (%) of studies with at least one result suggesting an effect at *p* < 0.05	4 (33)	9 (82)	10 (71)	17 (77)
Median no. (range) of outcomes reported	17.5 (4–31)	10 (1–80)	16 (9–44)	7 (1–35)
Median no. (range) of outcomes suggesting an effect at *p* < 0.05	0 (0–6)	1.5 (0–7)	3 (0–15)	1.5 (0–12)
Abstract^a^	(*n* = 12)	(*n* = 11)	(*n* = 14)	(*n* = 20)
No. (%) of studies with at least one result suggesting a significant effect	4 (33)	7 (64)	10 (71)	15 (75)
Median no. (range) of outcomes reported	3.5 (1–36)	3 (1–5)	6.5 (3–44)	3 (1–64)
Median no. (range) of outcomes suggesting a significant effect	0 (0–6)	1 (0–3)	2 (0–5)	1.5 (0–7)
Authors’ interpretation of results [no. (%)]				
No effect of radiofrequency radiation	10 (83.3)	5 (45.5)	4 (28.6)	5 (22.7)
Effect of radiofrequency radiation	1 (8.3)	5 (45.5)	8 (57.1)	14 (63.6)
Unclear finding	1 (8.3)	1 (9)	2 (14.3)	3 (13.6)
Title [no. (%)]				
Neutral	7 (58)	5 (46)	8 (57)	17 (77)
Statement of effect	0 (0)	4 (36)	3 (21)	4 (18)
Statement of no effect	5 (42)	2 (18)	3 (21)	1 (5)

Percentages are column percentages.

a Two publications that did not report their source of funding had no abstracts.

**Table 3:** Probability of reporting at least one statistically significant result (*p* < 0.05) according to source of funding: crude and adjusted ORs (95% CIs) from logistic regression models.

	Source of funding				
	Industry (*n* = 12)	Public or charity (*n* = 11)	Mixed (*n* = 14)	Not reported (*n* = 22)	*p*-Value^a^
Crude	0.11 (0.02–0.78)	1 (reference)	0.56 (0.08–3.80)	0.76 (0.12–4.70)	0.04
Adjusted for					
No. of reported outcomes	0.12 (0.02–0.89)	1 (reference)	0.60 (0.08–4.28)	0.96 (0.15–6.23)	0.04
Median study size	0.08 (0.009–0.62)	1 (reference)	0.61 (0.08–4.59)	0.57 (0.08–4.02)	0.02
Study design (crossover, parallel, or other)	0.08 (0.01–0.68)	1 (reference)	0.38 (0.05–3.07)	1.16 (0.16–8.61)	0.029
Study quality					
Randomization adequate	0.04 (0–0.56)	1 (reference)	0.16 (0.01–2.15)	1.27 (0.16–9.89)	0.005
Participants and assessors blinded	0.14 (0.02–0.96)	1 (reference)	0.54 (0.08–3.91)	0.76 (0.12–4.8)	0.09
Statistical analysis adequate	0.12 (0.02–0.85)	1 (reference)	0.67 (0.09–4.85)	0.54 (0.08–3.76)	0.07
Exposure setting appropriate	0.13 (0.02–0.89)	1 (reference)	0.47 (0.07–3.39)	0.86 (0.14–5.5)	0.06

Models adjusted for one variable at a time.

a From likelihood ratio tests.
